# Nature of Communicable Diseases

**Published:** 1877-12

**Authors:** 


### NATURE OF COMMUNICABLE DISEASES.

The views held by Dr. Richardson are so diametrically opposed to those of Prof. Lister and other advocates of the "germ theory" that I take the liberty of giving you a brief summary of the same.

Dr. Richardson in the begining stated that he entirely disbelieved in the theory of germs as possible or probable agents in the communication of disease; and, moreover, he had considerable doubts as to their existence. He holds that the poisons by which communicable diseases are spread abroad are the results of disturbed glandular action, and that they bear a general resemblance to snake-poison, which differs from them rather in being a natural product of the glands which form it than in any other way. According to this view, the poison of scarlet fever or of typhoid is not a germ, but simply a diseased secretion, indebted for any characters of vitality it may possess to the fact that it is a product, though a perverted one, of vital action. He described at length some of the peculiarities of the different specific poisons—different, he maintains, because elaborated by different organs—and traced many of the best-known laws of contagion to these peculiarities; as, for example, to the varying extent to which the poisons are affected by high or low temperatures. He regards the patient suffering from a communicable disease simply as a poison-producing animal, as one who is temporarily brought into a condition analogous to that of the poisonous snake, and who, as he returns to a state of health, casts off the poison by natural channels and ceases to produce more of it. It is, Dr. Richardson points out, a strong argument in favor of this view that the communicable diseases so frequently terminate in recovery. It is perfectly intelligible that the natural function of any gland may be perverted by disease, that it may produce a morbid secretion for a time, and that it may return to a natural condition; but if the body were infested with germs or other reproductive organisms, multiplying and occasioning illness by their presence, there is, he thinks, no intelligible reason why they should cease from multiplying as long as their host continues to live and to afford them the necessities of their existence. On the germ theory it is, he argued, difficult to understand how or why a patient ever should recover; but on his own view recovery is but one of the natural terminations of the derangement.

You can not afford me the space to attempt to weigh the opposed theories for which the evidence is as yet incomplete. Sanitary reformers, at any rate, may be expected to feel a bias in favor of a doctrine which, as opposed to the germ theory or the idea of the material of contagion as something full of independent life, is full of encouragement to their efforts. If, as Dr. Richardson argued so forcibly, the air around us is charged with invisible germs of disease, which come whence we know not, which have unlimited power of developement, and which never cease to multiply, what hope is there that the skill of man will ever overcome these hidden foes? If, on the other hand, the sick person is merely an animal rendered temporarily poisonous, we have only to isolate him from susceptible persons and to destroy the secretions which are the outlets of his poison, in order to render the spread of disease impossible. From this point of view the glandular theory, it must be confessed, encourages hope for the sanitary future of the human race.—*Louisville Medical News.*

